# The Influence of Oral Dydrogesterone and Vaginal Progesterone on Threatened Abortion: A Systematic Review and Meta-Analysis

**DOI:** 10.1155/2017/3616875

**Published:** 2017-12-17

**Authors:** Hee Joong Lee, Tae Chul Park, Jae Hoon Kim, Errol Norwitz, Banghyun Lee

**Affiliations:** ^1^Department of Obstetrics & Gynecology, College of Medicine, The Catholic University of Korea, Seoul, Republic of Korea; ^2^Department of Obstetrics & Gynecology, Gangnam Severance Hospital, Yonsei University College of Medicine, Seoul, Republic of Korea; ^3^Department of Obstetrics & Gynecology, Tufts University School of Medicine, Boston, MA, USA; ^4^Department of Obstetrics and Gynecology, Hallym University Kangdong Sacred Heart Hospital, Seoul, Republic of Korea

## Abstract

**Objective:**

To conduct systematic analyses to evaluate the efficacy of progesterone therapy for the prevention of miscarriages in pregnant women experiencing threatened abortion.

**Methods:**

In November 2016, we performed a systematic literature search and identified 51 articles in PubMed, Embase, and Cochrane databases. We identified nine randomized trials that included 913 pregnant women (including 322 treated with oral dydrogesterone, 213 treated with vaginal progesterone, and 378 control subjects) who met the selection criteria.

**Results:**

The incidence of miscarriage was significantly lower in the total progesterone group than in the control group (13.0% versus 21.7%; odds ratio, 0.53; 95% confidence interval (CI), 0.36 to 0.78; *P* = 0.001; *I*^2^, 0%). Moreover, the incidence of miscarriage was significantly lower in the oral dydrogesterone group than in the control group (11.7% versus 22.6%; odds ratio, 0.43; 95% CI, 0.26 to 0.71; *P* = 0.001; *I*^2^, 0%) and was lower in the vaginal progesterone group than in the control group, although this difference was nonsignificant (15.4% versus 20.3%; odds ratio, 0.72; 95% CI, 0.39 to 1.34; *P* = 0.30; *I*^2^, 0%). However, the incidence of miscarriage was not different between the oral dydrogesterone and vaginal progesterone groups.

**Conclusion:**

Progesterone therapy, especially oral dydrogesterone, can effectively prevent miscarriage in pregnant women experiencing threatened abortion.

## 1. Introduction

Progesterone maintains pregnancy by enhancing uterine quiescence [1]. During early pregnancy, the syncytiotrophoblast secretes human chorionic gonadotropin (hCG), which stimulates progesterone production in the corpus luteum by preventing regression of this tissue [2]. After seven to nine weeks of gestation, progesterone is directly secreted by the syncytiotrophoblast [2, 3]. Low serum hCG or progesterone levels may predict first trimester abortions [4]. During early pregnancy in women with threatened abortion, progesterone levels were lower in those who had a subsequent miscarriage than in those whose pregnancies continued to fetal viability [5]. Moreover, progesterone receptor antagonists may induce abortion or labor by increasing myometrial contractility and excitability throughout pregnancy [1, 6].

Threatened abortion, which occurs in 20% of all pregnancies, is diagnosed when vaginal bleeding with or without abdominal pain occurs during the first half of pregnancy. The required prerequisites for threatened abortion are a closed cervix and an intrauterine viable fetus [7, 8]. Unfortunately, nearly half of threatened abortions end in miscarriage [7, 8]. Progesterone has been used to treat threatened abortions, but its efficacy remains unclear [8–17].

Previous meta-analyses have shown that progesterone therapy may reduce the risk of miscarriage in pregnant women with threatened abortion. However, these meta-analyses were limited by a small number of included studies [8, 9]. Furthermore, these systematic analyses only included randomized studies that demonstrated the efficacy of the oral progesterone dydrogesterone, a pure progestin that was developed in the 1950s [8, 9, 18], and revealed that vaginal progesterone was ineffective [8, 9].

Although many studies have evaluated the impact of progesterone as a treatment for threatened abortion, only a few randomized studies have been conducted to explore this issue. Recently, some additional randomized studies reported the effect of progesterone therapy in pregnant women with threatened abortion. In this study, using an updated systematic analysis, we aimed to evaluate the effectiveness of progesterone therapy delivered via different administration routes for preventing miscarriages in pregnant women with threatened abortion.

## 2. Materials and Methods

### 2.1. Search Methods

In November 2016, we searched PubMed, Embase, and Cochrane databases for all relevant studies without limiting the publication year. A combination of the following terms using Boolean operators was used to perform the search: [(threatened abortion OR miscarriage) AND (progesterone OR progestin) AND randomized trial] and [(threatened abortion OR miscarriage) AND (dydrogesterone OR duphaston)]. Additional relevant studies that were not identified by the database searches were identified by examining the references of the selected clinical studies and review articles.

### 2.2. Selection Criteria

The following inclusion criteria were used for study selection: studies of pregnant women diagnosed with threatened abortion before 20 weeks of gestation, studies that compared any type of progesterone therapy with either placebo or conservative treatment, studies that compared different administration routes of progesterone therapy, studies that reported the incidence of miscarriage, and randomized or quasi-randomized controlled studies. The exclusion criteria were as follows: studies that were not case-match controlled, noncomparative studies, studies not in English, review articles, editorials, letters, case reports,* in vitro* research studies, and studies using other therapeutic agents. To avoid including duplicate information, when multiple studies were found to have included overlapping groups of patients, only the study with the largest number of events was included in the meta-analysis. Some results were published only in abstract form and not in full, and we found that some clinically useful evidence could be extracted from these studies.

### 2.3. Data Extraction and Outcomes of Interest

Two investigators developed a checklist for data recording, and they independently extracted the data of interest from the studies. If there was any disagreement between the findings of these investigators, they were resolved by discussion. The eligible population was classified into the following three groups: patients administered oral dydrogesterone therapy, patients administered vaginal progesterone therapy, and a control group that was administered placebo or conservative treatment. The following data were retrieved from the studies: the name of the first author, publication year, study design, eligibility criteria, sample size, interventions, and incidence of miscarriage. The incidence of miscarriage was the principal outcome of the meta-analysis and was compared among the treatment groups.

### 2.4. Overall Quality of the Body of Evidence

The quality of the evidence for the principle outcomes was evaluated using the Grading of Recommendations Assessment, Development, and Evaluation (GRADE) working group recommendations [21] as follows: the limitation (e.g., risk of bias) of the included studies, inconsistency of the observed effects, indirectness, imprecision, and risk of publication bias. The quality of the evidence was reported as follows: high quality, which indicates that further research is highly unlikely to change the confidence in the estimate of effect; moderate quality, which indicates that further research is likely to have an important impact on the confidence in the estimate of effect and may change the estimate; low quality, which indicates that further research is highly likely to have an important impact on the confidence in the estimate of effect and is likely to change the estimate; very low quality, which indicates that we are highly uncertain about the estimate.

### 2.5. Publication Bias and Statistical Analyses

To analyze the outcomes, a random-effects model was implemented using the Mantel-Haenszel method. The heterogeneity of the odds ratios (ORs) was assessed using the *I*^2^ statistic, and publication bias was identified using funnel plots. To generate a scatter plot, the horizontal axis was plotted as the OR of each study, and the vertical axis was plotted as the corresponding standard error of the log of the OR. Review Manager Version 5.3 software (The Nordic Cochrane Center, Copenhagen, Denmark) was used for the meta-analysis. GRADE evidence profiles were created using GRADEpro GDT. A *P* value of <0.05 indicated statistical significance. Subgroup analyses of the risk of miscarriage according to eligibility criteria, vaginal progesterone dose, and quality of studies were performed; however, a subgroup analysis based on oral dydrogesterone was not performed because similar doses were used in the studies (Table 1).

## 3. Results

### 3.1. Search Results and Characteristics and Assessments of the Risk of Bias in the Included Studies

**Figure 1:**
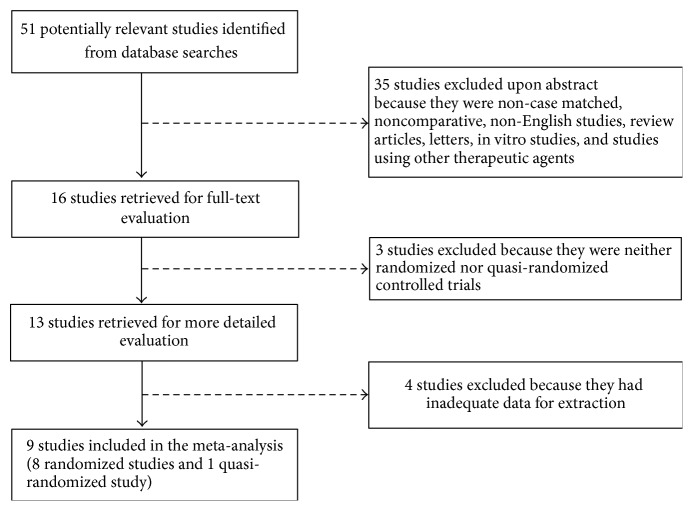
Flow chart of the procedure used for study selection.

**Figure 2:**
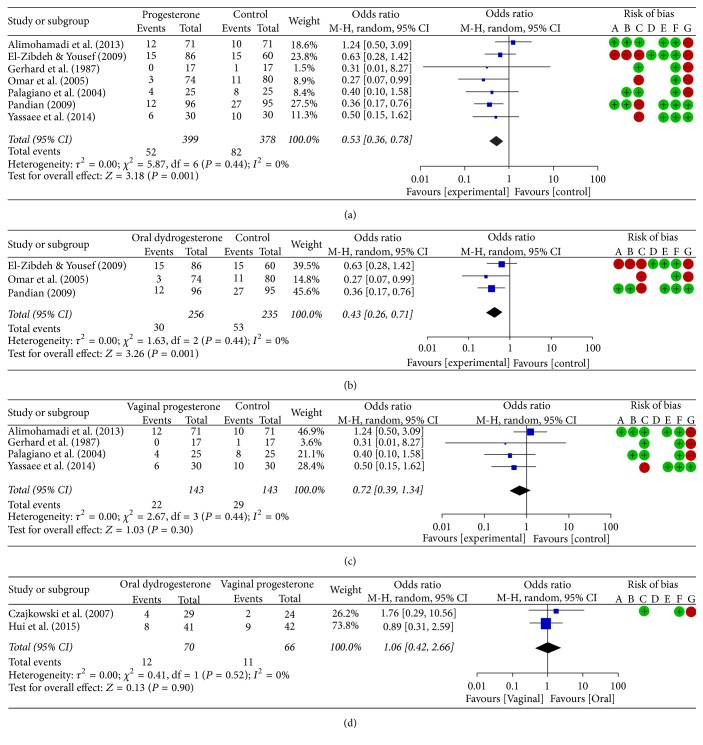
Forest plots and risk of bias: risk of miscarriage in pregnant women experiencing threatened abortion based on the route of progesterone administration. (a) Total progesterone versus control treatments. (b) Oral dydrogesterone versus control treatments. (c) Vaginal progesterone versus control treatments. (d) Oral dydrogesterone versus vaginal progesterone treatments. The risk of bias for each metric was assessed as low (+), high (−), or unclear (blank) for all the included studies as follows: A, random sequence generation (selection bias); B, allocation concealment (selection bias); C, blinding of the participants and personnel (performance bias); D, blinding of the outcome assessment (detection bias); E, incomplete outcome data (attrition bias); F, selective reporting (reporting bias); G, other bias. M-H, Mantel-Haenszel; CI, confidence interval.

Our literature search initially identified 51 potentially relevant studies; 8 randomized controlled studies and 1 quasi-randomized study that met the selection criteria were ultimately identified (Figure 1). The characteristics of the included studies are provided in Table 1, and assessments of the risk of bias in each study are provided in Table 2. Alimohamadi et al. [11] and Gerhard et al. [13] did not include information regarding the type (natural or synthetic) of vaginal progesterone that was administered. The study by Hui et al. [20] was only published in abstract form and did not provide information regarding the method for confirming live embryos or the dosages and duration of treatment with progestational agents. The included studies had a total of 913 pregnant women (including 322 treated with oral dydrogesterone, 213 treated with vaginal progesterone, and 378 control subjects) (Tables 1 and 3; Figure 2).

### 3.2. Risk of Miscarriage Based on the Route of Progesterone Administration in Pregnant Women Experiencing Threatened Abortion

The incidence of miscarriage was significantly lower in the total progesterone group than in the control group (13.0% versus 21.7%; odds ratio, 0.53; 95% confidence interval (CI), 0.36 to 0.78; *P* = 0.001; *I*^2^, 0%; 7 RCTs, 777 pregnant women; low quality evidence; Table 3(a), Figure 2(a), and Supplementary Figure 1(a)). Moreover, the incidence of miscarriage was significantly lower in the oral dydrogesterone group than in the control group (11.7% versus 22.6%; odds ratio, 0.43; 95% CI, 0.26 to 0.71; *P* = 0.001; *I*^2^, 0%; 3 RCTs, 491 pregnant women; low quality evidence; Table 3(a), Figure 2(b), and Supplementary Figure 1(b)) and was lower in the vaginal progesterone group than in the control group; however, this difference was not significant (15.4% versus 20.3%; odds ratio, 0.72; 95% CI, 0.39 to 1.34; *P* = 0.30; *I*^2^, 0%; 4 RCTs, 286 pregnant women; high quality evidence; Table 3(a), Figure 2(c), and Supplementary Figure 1(c)). However, the incidence of miscarriage was not different between the oral dydrogesterone and vaginal progesterone groups (17.1% versus 16.7%; odds ratio, 1.06; 95% CI, 0.42 to 2.66; *P* = 0.90; *I*^2^, 0%; 2 RCTs, 136 pregnant women; low quality evidence; Table 3(b), Figure 2(d), and Supplementary Figure 1(d)).

### 3.3. Subgroup Analyses

When comparing the subgroups based on eligibility criteria, the incidence of miscarriage among patients experiencing threatened abortion within 12 completed weeks of gestation was significantly lower in the total progesterone group than in the control group (*P* = 0.01). In patients experiencing threatened abortion before 20 weeks of gestation, the incidence of miscarriage was also lower in the total progesterone group than in the control group, although this difference was not significant (*P* = 0.20). When comparing the subgroups according to the vaginal progesterone dose (400 mg or less than 400 mg) because of the large discrepancy between the doses, high doses of progesterone were not associated with the incidence of miscarriage between the groups (*P* = 0.72). However, among the groups treated with a lower dose of hormone, the incidence of miscarriage was lower in the progesterone group than in the control group, although this difference was not significant (*P* = 0.14; Table 4 and Supplementary Figure 2).

## 4. Discussion

In this meta-analysis, we demonstrated that progesterone therapy may be effective in preventing miscarriages in pregnant women with threatened abortion. In particular, oral dydrogesterone prevented miscarriage in pregnant women more effectively than the control-treated groups (placebo or conservative treatment), although there was no difference between oral and vaginal progestational agents in preventing miscarriages in pregnant women experiencing threatened abortion.

The route of administration may influence the efficacy of progesterone therapy during pregnancy [22, 23]. Vaginal progesterone administration resulted in higher endometrial progesterone concentrations than those observed in patients administered oral and intramuscular progesterone [23]. Oral and vaginal administration routes are noninvasive, whereas intramuscular administration is invasive. Additionally, the oral and vaginal routes of administration are associated with acceptable and minimal side effects, respectively, whereas side effects were reported in one-third of pregnant women who received weekly intramuscular injections of progesterone to prevent recurrent preterm delivery [22–24]. Oral synthetic progestational agents, including dydrogesterone, have been developed to eliminate issues related to the variable bioavailability of natural formulations of oral progesterone [23]. A randomized study reported that micronized vaginal progesterone, but not oral dydrogesterone, decreased spiral artery pulsatility and the resistance index in the uteroplacental circulation of early pregnancies with threatened abortion [19].

In previous meta-analyses that included only randomized studies, vaginal and intramuscular progesterone administration effectively reduced the risk of preterm birth without any deleterious effects on fetal development [25, 26]. In a randomized study, a lower risk of preterm birth was associated with oral micronized progesterone than placebo [27]. Additionally, in a recent meta-analysis, oral dydrogesterone was as effective as vaginal progesterone for luteal phase support in assisted reproduction [28]. It has also been reported that intramuscular progesterone administration is associated with implantation, clinical pregnancy, and delivery rates that are comparable to those resulting from treatment with vaginal progesterone during stimulated IVF cycles [29]. These previous studies demonstrated that various progestational agents may induce similar outcomes despite the fact that differences in their efficacy were associated with the route of administration. In support of these studies, our meta-analysis showed that there was no difference in the rate of miscarriages between pregnant women with threatened abortion who were administered oral or vaginal progestational agents, although the small numbers of pregnant women and studies that were included limit the significance of these results.

Many studies have supported the efficacy of vaginal progesterone for preventing preterm births and luteal phase defects [25, 26, 28, 29]. Therefore, it is possible that miscarriages in pregnant women with threatened abortion might also be prevented by vaginal progesterone. However, a previous meta-analysis that included a small number of randomized studies showed that oral dydrogesterone, but not vaginal progesterone, reduced the incidence of miscarriage in pregnant women with threatened abortion [9]. Although we included a few additional recently reported randomized studies in our meta-analysis, the number of studies analyzed remained small. Our study also failed to show that vaginal progesterone was more effective in preventing miscarriages in pregnant women with threatened abortion than that in the controls, although we did find that oral dydrogesterone was effective. However, based on the subgroup analyses, our study showed that progesterone therapy was effective in preventing miscarriage—especially in pregnant women experiencing threatened abortion during the first trimester of pregnancy. This meta-analysis clearly showed the effectiveness of progesterone therapy for the prevention of miscarriage. These findings indicate that well-designed and large-scale studies are necessary to further demonstrate impact of progesterone therapy.

Our meta-analysis had several limitations. First, only studies that were either randomized or quasi-randomized and evaluated either oral dydrogesterone or vaginal progesterone administration were included in this analysis. Unfortunately, there were neither randomized nor quasi-randomized trials that evaluated the efficacy of intramuscular progesterone administration or oral formulations of progestins other than dydrogesterone in pregnant women experiencing threatened abortion. Second, because there is a paucity of studies that provided adequate data, we included small-scale studies as well as those with poor methodological quality in our analysis. Third, in the analyses comparing efficacy between oral progesterone and control treatments, between vaginal progesterone and control treatments, and between oral and vaginal progesterone, only a few eligible studies that included a small cohort of pregnant women could be analyzed. Finally, our searches were limited to the studies published in English. We found 2 studies not written in English that met our eligibility criteria. However, the significance of those studies was limited based on the publication year (1967) and lack of accessibility (no available abstract in English and difficulty finding experts in the relevant languages).

In conclusion, based on our systematic review and meta-analysis, we suggest that progesterone therapy, especially oral dydrogesterone, may effectively prevent miscarriages in pregnant women with threatened abortion. Although the number, scale, and methodological quality of the eligible studies limit the significance of our meta-analysis results, these results are important because we systemically analyzed all currently available randomized studies. Large-scale, multicenter, randomized and controlled studies are needed to better evaluate the efficacy of progesterone therapy in pregnant women with threatened abortion.

## Figures and Tables

**Table 1:** Characteristics of the included studies (*n* = 9).

Study	Year	Study design	Eligibility criteria	Sample size	Interventions

Alimohamadi et al. [11].	2013	Randomized (double-blind)	Vaginal bleeding and uterine cramps before the 20th week of pregnancy, live singleton by ultrasound	71 71	*Vaginal progesterone*: 200 mg, twice a day for 1 week *Control*: placebo using the same method

Czajkowski et al. [19].	2007	Randomized (double-blind)	Vaginal bleeding usually accompanied by abdominal pain before 12 weeks of pregnancy, live singleton by ultrasound	29 24	*Vaginal progesterone*: micronized, 300 mg, once per day for 6 weeks^a^ *Oral dydrogesterone*: 30 mg using the same method

El-Zibdeh and Yousef [12].	2009	Quasi-randomized (open-label)	Mild or moderate vaginal bleeding during the first trimester of pregnancy, live embryo by ultrasound	86 60	*Oral dydrogesterone*: 10 mg, twice per day until 1 week after bleeding had stopped *Control*: conservative treatment

Gerhard et al. [13].	1987	Randomized (double-blind)	Vaginal bleeding during the first trimester of pregnancy, live singleton by ultrasound	17 17	*Vaginal progesterone*: 25 mg, twice per day for 14 days after bleeding had stopped *Control*: placebo using the same method

Hui et al. [20].^b^	2015	Randomized	Vaginal bleeding between weeks 6 and 10 of pregnancy	41 42	*Vaginal progesterone*: micronized *Oral dydrogesterone*

Omar et al. [14].	2005	Randomized (open-label)	Mild or moderate vaginal bleeding before 13 weeks of pregnancy, live embryo by ultrasound	74 80	*Oral dydrogesterone*: initial: 40 mg; maintenance: 10 mg, twice per day until bleeding had stopped or for 1 week^c^ *Control*: conservative treatment

Pandian [15].	2009	Randomized (open-label)	Vaginal bleeding up to the 16th week of pregnancy, live embryo by ultrasound	96 95	*Oral dydrogesterone*: initial: 40 mg; maintenance: 10 mg, twice per day until the 16th week of pregnancy *Control*: conservative treatment

Palagiano et al. [16].	2004	Randomized (double-blind)	Vaginal bleeding and uterine cramps between weeks 6 and 12 of pregnancy with a previous diagnosis of inadequate luteal phase, live embryo by ultrasound	25 25	*Vaginal progesterone*: micronized, 90 mg, once per day for 5 days^d^ *Control*: placebo using the same method

Yassaee et al. [17].	2014	Randomized (single-blind)	Vaginal bleeding until the 20th week of pregnancy, live singleton by ultrasound	30 30	*Vaginal progesterone*: micronized, 400 mg, once per day until bleeding stopped within less than 1 week *Control*: conservative treatment^e^

^a^Adamed Inc., Poland; ^b^limited information was available because the study was published only in abstract form; ^c^unclear data regarding the duration of treatment; ^d^Crinone 8%® (progesterone gel, Merck Serono Inc., Germany); ^e^Cyclogest® (Actavis Inc., UK).

**Table 2:** Assessments of the risk of bias in the included studies.

Study	Random sequence generation (selection bias)	Allocation concealment (selection bias)	Blinding of the participants and personnel (performance bias)	Blinding of outcome assessment (detection bias)	Incomplete outcome data (attrition bias)	Selective reporting (reporting bias)	Other bias
Alimohamadi et al. [11].							

Authors' judgement	Low risk	Low risk	Low risk	Unclear	Low risk	Low risk	High risk
Support for judgement	Adequate method for randomization	Adequate allocation concealment	Blinding of the participants and personnel	No information for blinding of outcome assessors	No incomplete outcome data	Report of all outcomes	No intention to treat analysis

Czajkowski et al. [19].							

Authors' judgement	Unclear	Unclear	Low risk	Unclear	Unclear	Low risk	High risk
Support for judgement	No information regarding the method used for randomization	No mention for the method of allocation concealment	Blinding of the participants and personnel	No information for blinding of outcome assessors	No information for the number of participants who were lost for follow-up according to subgroups	Report of all outcomes	No intention to treat analysis

El-Zibdeh and Yousef [12].							

Authors' judgement	High risk	High risk	High risk	Low risk	Low risk	Low risk	High risk
Support for judgement	Quasi-randomized participants based on which day of the week the pregnant women came to the clinic	No allocation concealment	Neither blinding of the participants nor personnel	Adequate blinding for outcome assessors	No incomplete outcome data, analysis in all participants enrolled	Report of all outcomes	No intention to treat analysis for some variables

Gerhard et al. [13].							

Authors' judgement	Unclear	Unclear	Low risk	Unclear	Unclear	Low risk	High risk
Support for judgement	No information regarding the method used for randomization	No mention for the method of allocation concealment	Blinding of the participants and personnel	No information for blinding of outcome assessors	No information for the number of participants who were lost for follow-up according to subgroups	Report of all outcomes	No intention to treat analysis

Hui et al. [20].							

Authors' judgement	Unclear	Unclear	Unclear	Unclear	Unclear	Unclear	Unclear
Support for judgement	No information regarding the method used for randomization	No mention for the method of allocation concealment	No information	No information	No information	No information	No information

Omar et al. [14].							

Authors' judgement	Unclear	Unclear	High risk	Unclear	Unclear	Low risk	High risk
Support for judgement	No information regarding the method used for randomization	No mention for the method of allocation concealment	Neither blinding of the participants nor personnel	No information for blinding of outcome assessors	No information for the number of participants who were lost for follow-up according to subgroups	Report of all outcomes	No intention to treat analysis

Pandian [15].							

Authors' judgement	Low risk	Low risk	High risk	Unclear	Low risk	Low risk	Low risk
Support for judgement	Adequate method for randomization	Adequate allocation concealment	Neither blinding of the participants nor personnel	Unclear information for blinding of outcome assessors	No incomplete outcome data, analysis in all participants enrolled	Report of all outcomes	No additional bias

Palagiano et al. [16].							

Authors' judgement	Unclear	Low risk	Low risk	Unclear	Unclear	Low risk	High risk
Support for judgement	No information regarding the method used for randomization	Adequate allocation concealment	Blinding of the participants and personnel	No information for blinding of outcome assessors	No information for the number of participants who were lost for follow-up	Report of all outcomes	No intention to treat analysis

Yassaee et al. [17].							

Authors' judgement	Unclear	Unclear	High risk	Unclear	Low risk	Low risk	Low risk
Support for judgement	No information regarding the method used for randomization	No mention for the method of allocation concealment	No blinding of the participants	No information for blinding of outcome assessors	No incomplete outcome data, analysis in all participants enrolled	Report of all outcomes	No additional bias

**Table:** (a) Progesterone agents versus control treatments

Quality assessment							Number of patients (%)		Absolute effect (95% CI)		Relative effect (95% CI)	Quality	Importance
No. of studies	Study design	Risk of bias	Inconsistency	Indirectness	Imprecision	Publication bias	Progesterone agents	Control	Progesterone agents	Control			
Outcome: miscarriage													

Progesterone versus control													

7	Randomized trials	Serious^a^	Not serious	Not serious	Serious^b^	None	52/399 (13.0)	82/378 (21.7)	128 per 1000	217 per 1000	*OR 0.53* (0.36 to 0.78)	*⨁⨁*◯◯ Low	Critical
									*89 fewer per 1,000* (from 39 fewer to 126 fewer)				

Oral dydrogesterone versus control													

3	Randomized trials	Serious^a^	Not serious	Not serious	Serious^b^	None	30/256 (11.7)	53/235 (22.6)	112 per 1000	226 per 1000	*OR 0.43* (0.26 to 0.71)	*⨁⨁*◯◯ Low	Critical
									*114 fewer per 1,000* (from 54 fewer to 155 fewer)				

Vaginal progesterone versus control													

4	Randomized trials	Not serious	Not serious	Not serious	Not serious	None	22/143 (15.4)	29/143 (20.3)	155 per 1000	203 per 1000	*OR 0.72* (0.39 to 1.34)	*⨁⨁⨁⨁* High	Critical
									*48 fewer per 1,000* (from 51 more to 113 fewer)				

CI: confidence interval; OR: odds ratio; ^a^either the participants or personnel were not blinded in these studies (performance bias); ^b^the 95% CI includes appreciable harm or benefit.

**Table:** (b) Oral dydrogesterone versus vaginal progesterone

Quality assessment							Number of patients (%)		Absolute effect (95% CI)		Relative effect (95% CI)	Quality	Importance
No. of studies	Study design	Risk of bias	Inconsistency	Indirectness	Imprecision	Publication bias	Oral dydrogesterone	Vaginal progesterone	Oral dydrogesterone	Vaginal progesterone			
Outcome: miscarriage													

2	Randomized trials	Serious^c^	Not serious	Not serious	Serious^c^	None	12/70 (17.1)	11/66 (16.7)	175 per 1000	167 per 1000	*OR 1.06* (0.42 to 2.66)	*⨁⨁*◯◯ Low	Critical
									*8 more per 1,000* (from 89 fewer to 181 more)				

CI: confidence interval; OR: odds ratio; ^c^limited information was available because a study was only published in abstract form.

**Table 4:** Subgroup analyses of risk of miscarriage according to eligibility criteria and vaginal progesterone dose.

Subgroups	Studies, *n*	Number of patients (%)		OR (95% CI)	*P* value	Heterogeneity (*I*^2^)
		Progesterone	Control			
*Eligibility criteria*						
Threatened abortion within 12 completed weeks of gestation	4 (12,13,14,16)	22/202 (10.9)	35/182 (19.2)	0.47 (0.26–0.86)	0.01	0%
Threatened abortion before 20 weeks of gestation	3 (11,15,17)	30/197 (15.2)	47/196 (24.0)	0.60 (0.27–1.31)	0.20	53%
*Vaginal progesterone dose*						
High^a^	2 (11,17)	18/101 (17.8)	20/101 (19.8)	0.85 (0.35–2.05)	0.72	30%
Low^b^	2 (13,16)	4/42 (9.5)	9/42 (21.4)	0.39 (0.11–1.37)	0.14	0%

^a^High-dose use of vaginal progesterone included studies that administered 400 mg per day for 1 week or until bleeding stopped within less than 1 week. ^b^Low-dose use of vaginal progesterone included studies using a dose lower than the reported high dose.

## Supplementary Materials

Supplementary 1 Supplementary Figure  1: Funnel plots: risk of miscarriage in pregnant women experiencing threatened abortion based on the route of progesterone administration.

Supplementary 2 Supplementary Figure  2: Funnel plots: subgroup analyses of risk of miscarriage according to eligibility criteria, vaginal progesterone dose, and the study quality.
