# Supplementary material for: The Influence of Oral Dydrogesterone and Vaginal Progesterone on Threatened Abortion: A Systematic Review and Meta-Analysis
Source: Biomed Res Int. 2017 Dec 17;2017:3616875. doi: 10.1155/2017/3616875 (PMC5748117; doi:10.1155/2017/3616875)
Supplement: Supplementary 1 — Supplementary Figure 1: Funnel plots: risk of miscarriage in pregnant women experiencing threatened abortion based on the route of progesterone administration. [file 3616875.f1.docx]

**Supplementary FIGURE 1:** Funnel plots: Risk of miscarriage in pregnant women experiencing threatened abortion based on the route of progesterone administration.

**(a)**

**(c)**

**(b)**

**(d)**


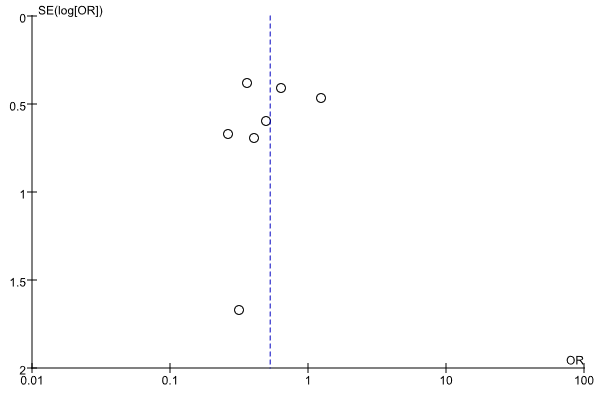

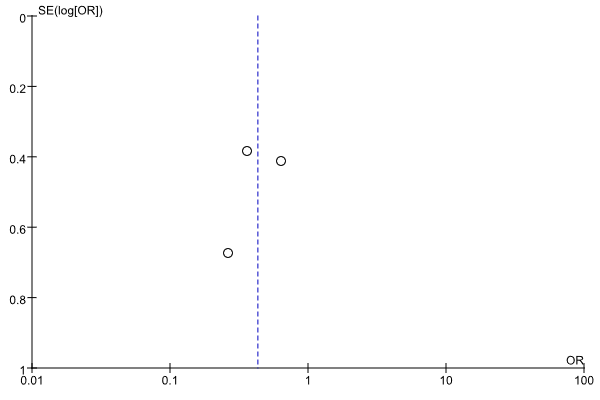

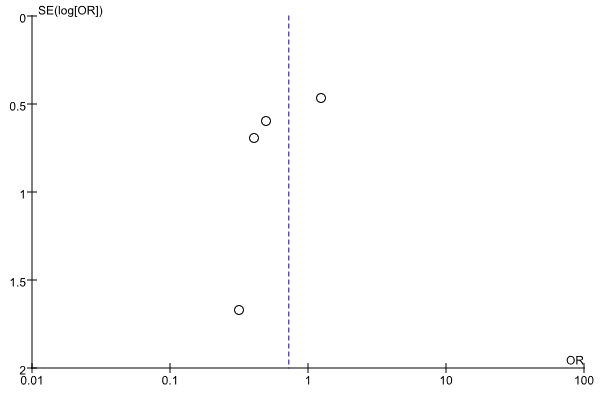

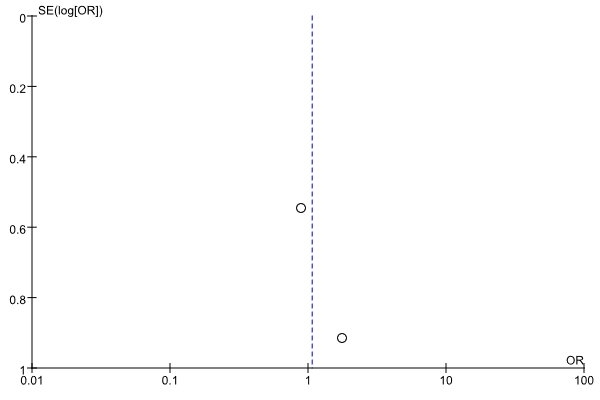


(a) Total progesterone vs control treatments. (b) Oral dydrogesterone vs control treatments. (c) Vaginal progesterone vs control treatments. (d) Oral dydrogesterone vs vaginal progesterone treatments
