# Supplementary material for: The Influence of Oral Dydrogesterone and Vaginal Progesterone on Threatened Abortion: A Systematic Review and Meta-Analysis
Source: Biomed Res Int. 2017 Dec 17;2017:3616875. doi: 10.1155/2017/3616875 (PMC5748117; doi:10.1155/2017/3616875)
Supplement: Supplementary 2 — Supplementary Figure 2: Funnel plots: subgroup analyses of risk of miscarriage according to eligibility criteria, vaginal progesterone dose, and the study quality. [file 3616875.f2.docx]

**Supplementary FIGURE 2:** Funnel plots: Subgroup analyses of risk of miscarriage according to eligibility criteria, vaginal progesterone dose, and the study quality.

**(a)**

**(c)**

**(b)**

**(d)**


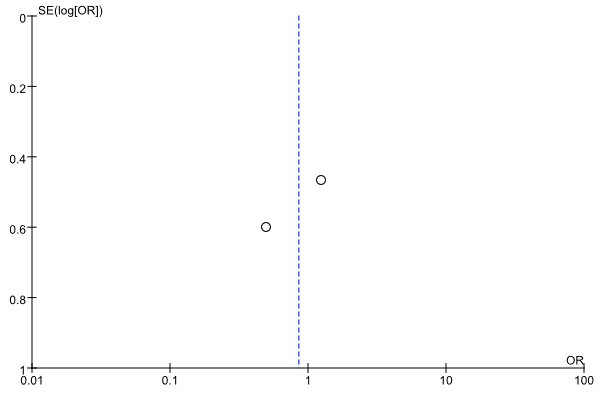

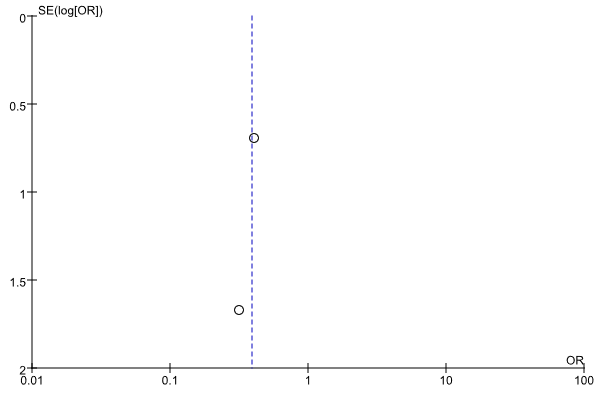

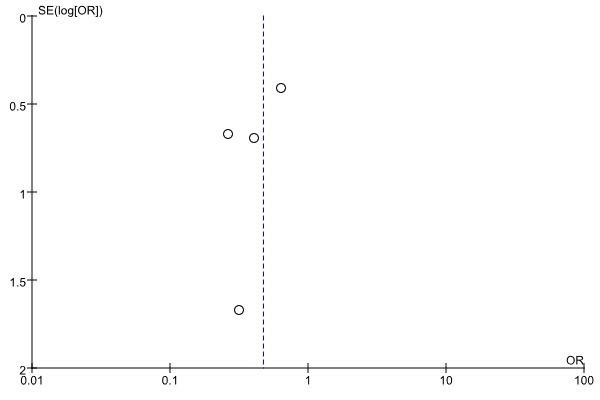

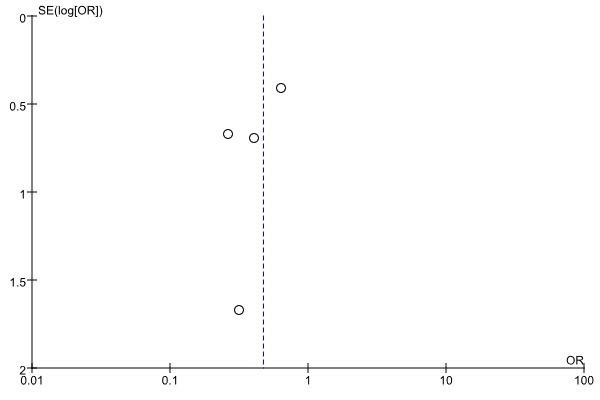


(a) Threatened abortion within 12 completed weeks of gestation. (b) Threatened abortion before 20 weeks of gestation. (c) High-dose use of vaginal progesterone. (d) Low-dose use of vaginal progesterone.
